# An engineered cryptic Hxt11 sugar transporter facilitates glucose–xylose co-consumption in *Saccharomyces cerevisiae*

**DOI:** 10.1186/s13068-015-0360-6

**Published:** 2015-11-02

**Authors:** Hyun Yong Shin, Jeroen G. Nijland, Paul P. de Waal, René M. de Jong, Paul Klaassen, Arnold J. M. Driessen

**Affiliations:** Molecular Microbiology, Groningen Biomolecular Sciences and Biotechnology, University of Groningen, Zernike Institute for Advanced Materials and Kluyver Centre for Genomics of Industrial Fermentation, Nijenborgh 7, 9747 AG Groningen, The Netherlands; DSM Biotechnology Center, Alexander Fleminglaan 1, 2613 AX Delft, The Netherlands

**Keywords:** Sugar transport, Directed evolution, Lignocellulose conversion, Yeast

## Abstract

**Background:**

The yeast *Saccharomyces cerevisiae* is unable to ferment pentose sugars like d-xylose. Through the introduction of the respective metabolic pathway, *S. cerevisiae* is able to ferment xylose but first utilizes d-glucose before the d-xylose can be transported and metabolized. Low affinity d-xylose uptake occurs through the endogenous hexose (Hxt) transporters. For a more robust sugar fermentation, co-consumption of d-glucose and d-xylose is desired as d-xylose fermentation is in particular prone to inhibition by compounds present in pretreated lignocellulosic feedstocks.

**Results:**

Evolutionary engineering of a d-xylose-fermenting *S. cerevisiae* strain lacking the major transporter *HXT1*–*7* and *GAL2* genes yielded a derivative that shows improved growth on xylose because of the expression of a normally cryptic *HXT11* gene. Hxt11 also supported improved growth on d-xylose by the wild-type strain. Further selection for glucose-insensitive growth on d-xylose employing a quadruple hexokinase deletion yielded mutations at N366 of Hxt11 that reversed the transporter specificity for d-glucose into d-xylose while maintaining high d-xylose transport rates. The Hxt11 mutant enabled the efficient co-fermentation of xylose and glucose at industrially relevant sugar concentrations when expressed in a strain lacking the *HXT1*–*7* and *GAL2* genes.

**Conclusions:**

Hxt11 is a cryptic sugar transporter of *S. cerevisiae* that previously has not been associated with effective d-xylose transport. Mutagenesis of Hxt11 yielded transporters that show a better affinity for d-xylose as compared to d-glucose while maintaining high transport rates. d-glucose and d-xylose co-consumption is due to a redistribution of the sugar transport flux while maintaining the total sugar conversion rate into ethanol. This method provides a single transporter solution for effective fermentation on lignocellulosic feedstocks.

**Electronic supplementary material:**

The online version of this article (doi:10.1186/s13068-015-0360-6) contains supplementary material, which is available to authorized users.

## Background

Currently, most of the industrial materials and fuels are derived from petroleum that with time will become depleted [1]. Lignocellulosic biomass, the most abundant raw material from hardwood, softwood, and agricultural residues, is considered as an abundant potential feedstock for the future production of chemicals and biofuels [2]. Successful conversion of cellulosic biomass into biofuel will require organisms capable of the efficient utilization of xylose, because the free sugar content of plant biomass hydrolysates amounts to ~70 % glucose and ~30 % of xylose [3]. In current fermentation processes, *Saccharomyces cerevisiae* is the key microorganism for ethanol production as it has the natural capability to convert glucose into ethanol. However, this yeast is unable to ferment xylose because it does not possess a functional xylose dissimilation pathway. Previous research has shown that various metabolic engineering strategies can be employed to express both fungal and bacterial genes encoding xylose-utilizing pathways in *S. cerevisiae* [4, 5]. A xylose-utilizing pathway in *S. cerevisiae* has for instance been realized by the introduction of the genes encoding xylose reductase and xylitol dehydrogenase isolated from fungal species [6] or the introduction of heterologous xylose isomerases resulting in a good ethanol yield and productivity during xylose fermentation [7]. Still, in this process, xylose consumption is slow as long as there is residual glucose present in the fermentation broth.

Efficient utilization of xylose is a prerequisite for the large-scale production of biofuels. During pretreatment, inhibitors such as furans, phenols, carboxylic and inorganic acids, aldehydes and alcohols are released from lignocellulosic biomass and these compounds inhibit xylose fermentation [8, 9]. For instance, acetic acid has a strong and specific negative impact on the xylose consumption rate, which, after glucose depletion, dramatically slows down leaving up to 50 % of the xylose unused [10]. Therefore, growth on xylose in the second phase is more profoundly inhibited by monocarboxylic organic acids compared to growth on glucose. Moreover, engineered *S. cerevisiae* strains show a reduced ethanol tolerance during xylose fermentation as compared to glucose fermentation [11]. Thus, co-fermentation of xylose with glucose would be beneficial for ethanol production from lignocellulosic hydrolysate and a necessity for the development of a robust process.

One of the bottlenecks in the conversion of lignocellulosic biomass to fuels and chemicals is efficient sugar transport [12, 13]. Inefficient transport of pentoses, in addition to low tolerance and throughput, prevent large-scale industrial production of next-generation biofuels [14, 15]. Whereas *S. cerevisiae* contains a multitude of hexose transporters that mediate the uptake of glucose [13], it lacks specific pentose transporters. Instead, pentoses such as xylose enter the cell via the endogenous hexose transporters. This is a sub-optimal process as xylose transport is of low affinity. Importantly, in raw feedstocks, glucose is an abundant sugar and it effectively competes with pentoses for uptake. Engineered strains capable of xylose metabolism indeed first utilize the glucose, and once this sugar is depleted, xylose conversion occurs, resulting in a long fermentation process and an overall slow and incomplete conversion. To improve the xylose uptake activity of *S. cerevisiae*, it is necessary to identify specific transporters mediating the uptake of xylose. One approach is to express specific xylose transporters from other organisms in a xylose-fermenting *S. cerevisiae*. However, this approach has met little success and only led to minor improvements in the growth rate on xylose and ethanol production [16, 17]. A main problem with heterologous expression of xylose transporters is inefficient targeting to the plasma membrane. Often, overexpressed transporters enter a cellular degradation pathway ending up in vacuoles. A more favorable solution is to employ the yeast endogenous arsenal of transporters and identify those systems with an improved ability to transport xylose. Such transporters would remain connected to the yeast regulatory network that presents different glucose transporters at the cell surface depending on the physiological state of the cells and the glucose concentration in the medium [18].

*S. cerevisiae* contains at least eight hexose transporters (Hxt) that mediate glucose uptake [19]. Although classified as specific glucose transporters, several Hxt proteins also mediate uptake of xylose albeit with lower affinity [6, 20]. A multiple deletion strain lacking the key transporters Hxt1–Hxt7 and Gal2 was shown to be unable to grow on xylose, while poor growth on glucose was observed [21]. In that study, the remaining Hxt8–Hxt17 transporters were not investigated for xylose uptake as the transcription of their genes is either very low or absent. Nevertheless, these systems present a so far untapped source of transporters with potential xylose transport activity.

Here, we report on an evolutionary engineering approach using chemostat cultivation of a xylose-fermenting *S. cerevisiae* strain in which the main *HXT1*–*7* and *GAL2* transporter genes were deleted. Because of the deficiency in xylose transport, this strain is unable to grow on xylose. However, long-term chemostat culturing allowed cells to adapt and grow on xylose, which could be attributed to the expression of the cryptic *HXT11* gene that so far has not been implicated in xylose transport. Further engineering of Hxt11 yielded variants with a dramatically improved specificity towards xylose allowing co-consumption of xylose and glucose at industrial relevant concentrations.

## Results and discussion

### Evolutionary engineering of *S. cerevisiae* strain DS68625 using a chemostat

**Fig. 1:**
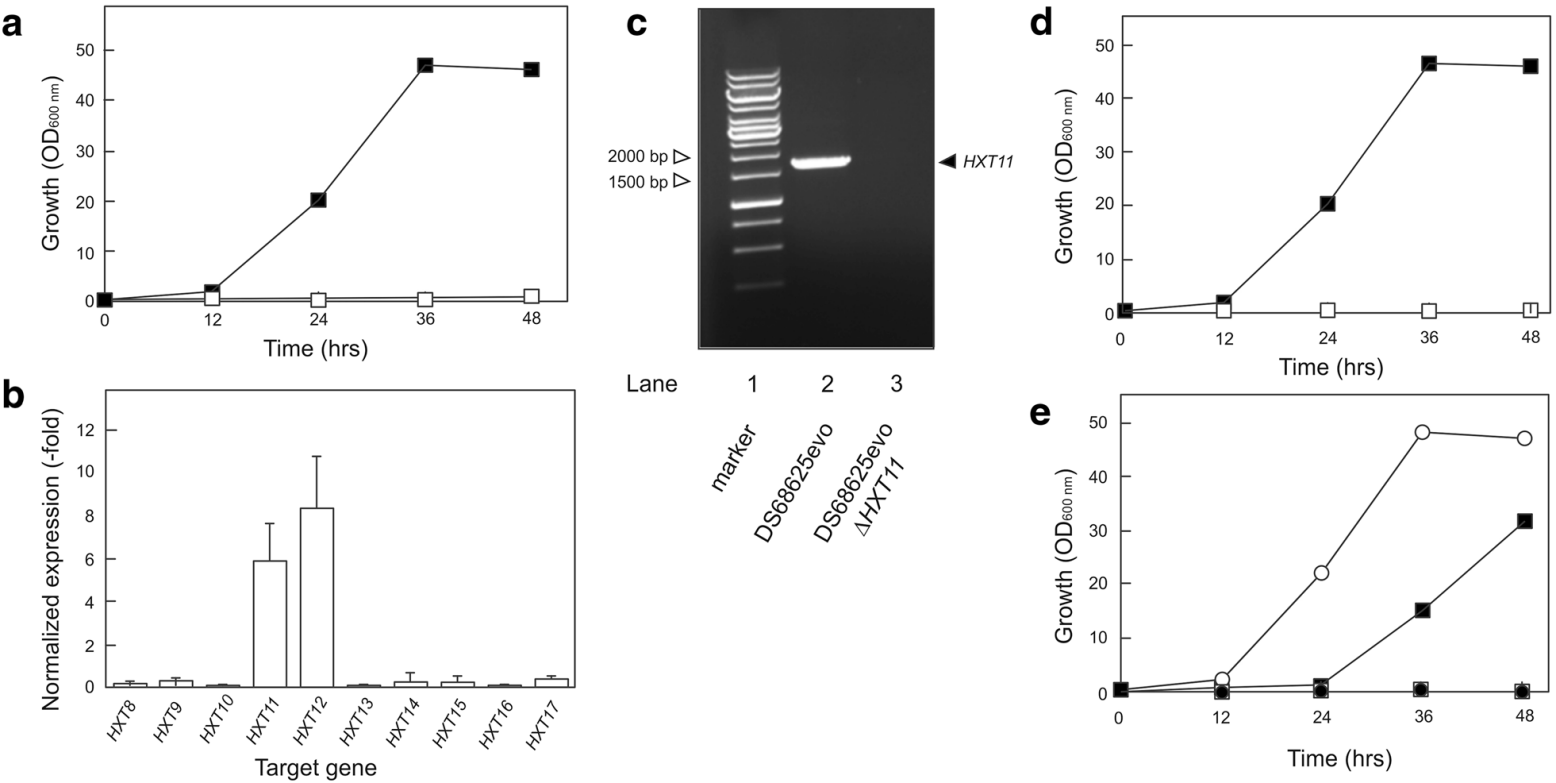
Utilization of d-xylose by up-regulation of the cryptic Hxt11 hexose transporter. **a** Growth curve of evolved strain DS68625-evo (*filled square*) and wild-type DS68625 (*open square*) on 2 % d-xylose. **b** Expression level of *HXT 8*–*17* in the strain DS68625-evo. The *error bars* represent the standard error of the mean from two technical samples. **c** Deletion of the *HXT11* gene from the strain DS68625-evo. **d** Growth of the DS68625evo (*filled square*) and ∆*hxt11* DS68625-evo (*open square*) strains on 2 % d-xylose. **e** Growth of strain DS68616 containing the natively expressed set of Hxt transporters (*filled square*), DS68625 lacking *HXT1*–*7* and *GAL2* but expressing *HXT11* (*open circle*) and *HXT12* (*filled circle*), and the empty vector control (*open square*) on 2 % d-xylose

*S. cerevisiae* DS68616 is a xylose-fermenting yeast that contains an engineered xylose pathway based on a xylose isomerase and xylulose kinase that shuttle the xylose into the pentose phosphate pathway [22]. A derivative of *S. cerevisiae* DS68616 was constructed in which the *HXT1*–*7* and *GAL2* genes that specify the main hexose transporters were deleted (Table 1). As these hexose transporters are responsible for xylose uptake by the cells, the resultant DS68625 strain is unable to grow on xylose alone (Fig. 1a) [19, 23]. This strain was used to initiate an evolution experiment in continuous culture under anaerobic condition using xylose as limiting carbon source. Cells were grown at various dilution rate of 0.01–0.05 h^−1^, and the feed initially contained 2 % xylose and 0.1 % maltose as carbon sources which was changed to 2 % xylose as the sole carbon source after more than 300 h. Maltose was used to support growth of strain DS68625 in the initial stages of the evolution experiment. After 1000 h (~31 generations), cells capable of growing on 2 % xylose were transferred to shake flask cultures and the single colony isolate growing the most efficiently on xylose was selected. This yielded the evolved strain DS68625-evo that was able to grow on 2 % xylose medium (Fig. 1a), whereas the original DS68625 strain showed no growth on xylose, validating the evolved phenotype.

**Table 1:** Strains and plasmids

Strain/plasmid	Relevant genotype and/or characteristics	Source or reference
*S. cerevisiae* strains		
DS68616	*Mat a, ura3*-*52, leu2*-*112, gre3::loxP, loxP*-*Ptpi:TAL1, loxP*-*Ptpi::RKI1, loxP*-*Ptpi*-*TKL1, loxP*-*Ptpi*-*RPE1, delta::Padh1XKS1Tcyc1*-*LEU2, delta::URA3*-*Ptpi*-*xylA*-*Tcyc1, delta::his3*	This paper
DS68625	DS68616, *his3::loxP, hxt2::loxP*-*kanMX*-*loxP, hxt367::loxP*-*hphMX*-*loxP, hxt145::loxP*-*natMX*-*loxP, gal2::loxP*-*zeoMX*-*loxP*	This paper
DS68625-evo	DS68625-derivative after evolutionary engineering	This paper
DS71054	DS71055, *glk1*::*lox*72; *hxk1*::*lox*P; *hxk2:*:*lox*72; *gal1:*:*lox*P; *his3*::loxPnatMXloxP	This paper
DS71055	DS68616-derivative after evolutionary engineering	DSM, The Netherlands
Plasmids		
pRS313	*E. coli*/yeast shuttle vector; *CEN6, ARSH4, HIS3,* Amp^r^	[27]

### Expression of HXT genes in the evolved strain DS68625-evo

Of the 20 members of the *HXT* gene family, only *HXT1*–*HXT7* are known to encode functional glucose transporter with various affinities for xylose, while *HXT8*–*HXT17* are not expressed when cells are grown in the presence of glucose [24]. Previous studies suggested that the remaining 10 *HXT* genes (*HXT8*–*17*) are either not expressed or are insufficiently expressed to support growth on xylose [6, 25, 26]. We hypothesized that growth restoration on xylose by the evolved DS68625-evo strain is due to the expression of one or more of the cryptic *HXT* genes (*HXT8*–*HXT17*). Importantly, PCR evidence indicated that in the evolved strain, *HXT1*–*7* and *GAL2* are still deleted, excluding a contamination of the chemostat experiment (data not shown). The expression patterns of *HXT8*–*17* in the evolved and progenitor strain were compared using cells incubated in a medium containing 2 % xylose. The transcription levels of *HXT11* and *HXT12* were increased by up to eightfold in the DS68625-evo strain at the beginning of exponential growth phase compared to DS68625, whereas the other *HXT* genes (i.e., *HXT8*–*HXT10*, and *HXT13*–*17*) were repressed (Fig. 1b). This suggests that expression of *HXT11* and/or *HXT12* causes the rescued growth phenotype on xylose.

### Hxt11 is responsible for xylose uptake in the evolved strain DS68625-evo

Hxt11 and Hxt12 are very homologous membrane proteins (i.e., about 98 %) while *HXT12* is considered a pseudo-gene in the S288c genome due to a frame shift mutation [6]. *HXT11* and *HXT12* were cloned into the low-copy yeast expression vector pRS313-P7T7 under control of the truncated HXT7 promoter [27]. This allows constitutive expression of genes in cells grown on minimal medium containing glucose and/or xylose. The corresponding vectors were transformed into strain DS68625 that is unable to consume xylose because of the aforementioned deficiency in transport. In contrast to *HXT12*, expression of *HXT11* supported growth of strain DS68625 on 2 % xylose (Fig. 1e). Remarkably, growth by the DS68625 strain expressing HXT11 on xylose was even improved relative to strain DS68616 containing the full complement of hexose transporters (Fig. 1e). Furthermore, growth and glucose utilization by the DS68625 strain expressing HXT11 were indistinguishable from that by strain DS68616 (Additional file 1: Figure S1) indicating that HXT11 is an efficient glucose transporter that can support high sugar fluxes. Next, the *HXT11* gene was inactivated in the DS68625-evo strain to ascertain that Hxt11 is the key determinant restoring growth of DS68625 on xylose (Fig. 1c). Strain DS68625-evo with the disrupted *HXT11* gene was no longer able to grow on xylose medium (Fig. 1d). This demonstrates that the spontaneous growth on xylose of the evolved DS68625-evo strain is due to the expression of *HXT11*.

To validate that growth restoration indeed is caused by increased xylose transport, uptake experiments were performed using [^14^C-] xylose. As a positive control, the *HXT2* gene was overexpressed in strain DS68625, as it has been shown to transport xylose with a reasonable affinity [20]. The strain expressing the Hxt11 transporter indeed showed the effective uptake of xylose with a low *K*_M_ of 84.2 mM and but high *V*_max_ of 84.6 nmol/mgDW min (Table 2). The *V*_max_ of Hxt11 for xylose uptake was about threefold higher as for Hxt2, while the affinity for xylose uptake was comparable. Compared to Hxt7 and Gal2, the affinity of Hxt11 for xylose uptake was more than twofold higher, while the Vmax of Hxt11 in the same range compared to that of Hxt7 and Gal2. In contrast, the affinity of Hxt11 for glucose is much lower than that of Hxt7 and Gal2 (Table 2) which makes Hxt11 an ideal scaffold for further engineering of its xylose specificity. In this respect, both with Hxt11 and Hxt2, [^14^C-] xylose uptake was strongly inhibited by glucose, but xylose transport by the Hxt11 transporter appeared less sensitive to glucose inhibition (Additional file 1: Figure S2).

**Table 2:** *K*_m_ and *V*_max_ values for d-glucose and d-xylose uptake by Hxt11 transporters expressed in strain DS68625

Transporter	*K*_m_ (mM)		*K*_m_ Glc/Xyl (ratio)	*V*_max_ (nmol/mg DW min)		Source
	Glucose	Xylose		Glucose	Xylose	
Hxt11	33.4 ± 2.1	84.2 ± 10.0	0.40	156.4 ± 7.6	84.6 ± 2.4	This study
Hxt11-N366D	87.0 ± 6.4	106.7 ± 21.7	0.82	197.8 ± 11.4	86.5 ± 2.0	This study
Hxt11-N366T	194.4 ± 47.9	46.7 ± 2.7	4.16	238.6 ± 7.4	76.2 ± 4.8	This study
Hxt11-N366 M	144.9 ± 36.0	50.1 ± 9.7	2.89	143.0 ± 17.2	65.0 ± 6.8	This study
Hxt2	n.d.	51.2 ± 0.1	n.d.	n.d.	23.8 ± 0.4	This study
Gal2	1.5 ± 0.2	225.6 ± 15.8	0.01	27.2 ± 0.9	91.3 ± 3.2	[36]
Hxt7	0.5 ± 0.1	200.3 ± 13.2	0	26.0 ± 1.1	67.0 ± 2.0	[36]

*n.d.* not determined, ± standard deviation with *n* = 2

### Genomic analysis of evolved strain DS68625-evo

The genome of the evolved strain DS68625-evo was sequenced and compared with that of the progenitor DS68625, resulting in the identification of 80 single-nucleotide polymorphisms (SNPs) in the DS68625-evo strain (Additional file 2: Table S1). To access which of the mutations is preliminary responsible for the changed expression level of the HXT11 gene, we focused on the SNPs that are related to HXT regulation. Of the 80 SNPs that resulted in amino acid changes, two are in genes that are potentially involved in such regulation. Gene *SIZ1* which encodes a small ubiquitin-like modifier (SUMO) is an important component of the regulation of the Snf1 function as it interacts with the SUMO-interacting sequence motif located near the active site of Snf1, which inactivates Snf1 protein directing it for destruction via the Slx5–Slx8 (SUMO-directed) ubiquitin ligase [28]. In the evolved strain, *SIZ1* carries two amino acid mutations, Asn531Tyr and Asn534Lys, which are not in a functionally defined region. In addition, a frame shift mutation was identified in gene *HAP4* at nucleotide 1619. Hap4p is a DNA-binding protein that is part of a multimeric HAP complex I that binds to the consensus 5′-TNATTGGT-3′ sequence in the promoter of target genes [29, 30] and affects gene expression. Possibly, these mutations contribute to the deregulation of *HXT11* gene expression observed in the evolved strain.

The exact mechanism how *HXT11* expression is induced in the evolved strain is unknown. As confirmed by genomic DNA sequencing, the nucleotide sequence of the promoter region of *HXT11* in the DS68625-evo strain was indistinguishable from that in the original DS68625 strain. Recently, it was reported that the expression of *HXT11* is positively regulated by the Pdr1p and Pdr3p transcriptional factors and that expression is not induced by glucose [31]. In addition, in xylose-grown cells, the genes encoding the enzymes of the citric acid and glyoxylate cycle, respiration and gluconeogenesis showed higher expression levels than in glucose repressed cells [32]. These genes are regulated by the transcriptional regulators *ADR1*, *CAT8*, *HAP4*, *SIP1*-*2* and -*4*, and *REG2* [33]. It seems that expression of *HXT11* is regulated differently from the main *HXT1*–*7* transporter genes, but the mechanism underlying expression of *HXT11* in the evolved strain is complex and likely not due to single mutations.

### Engineering of Hxt11 for improved xylose transport in the presence of glucose

To improve on the xylose transport specificity of Hxt11, the *HXT11* gene was mutagenized by error-prone PCR and subjected to a screen for improved xylose transport in *S. cerevisiae* in the presence of a high concentration of glucose. Herein, a strain was constructed that is unable to grow on d-glucose by deleting hexokinase activity-encoding genes in a strain that contains an engineered d-xylose metabolic pathway and therefore grows on d-xylose. The three hexokinases Hxk1p, Hxk2p and Glk1p differ in their affinity and specificity for glucose and ATP [34] and all phosphorylate glucose to glucose-6-phosphate. However, in an earlier study, the triple hexokinase deletion strain continued to grow on glucose after several rounds of evolutionary engineering, and re-sequencing of this evolved strain revealed a mutant *GAL1* sequence that rescued glucose phosphorylation in the triple hexokinase deletion strain thereby re-enabling growth on glucose [35]. Therefore, to enable a stable screening platform, four hexokinase genes, *GLK1*, *HXK1*, *HXK2* and *GAL1,* were deleted from *S. cerevisiae* strain DS71055 to block glucose consumption. The corresponding quadruple hexokinase deletion strain DS71054 (Table 1) is unable to grow on d-glucose but still grows on d-xylose (Additional file 1: Figure S3) and this growth phenotype did not revert when cells were exposed to xylose and glucose for longer periods of time (data not shown). The DS71054 strain is similar to the *S. cerevisiae* strain used by Farwick and coworkers [36], except that in the latter study, the putative hexokinase gene *YLR446* *W* was deleted instead of *GAL1*. In a previous study, *YLR446* *W* was found not to be responsible for the remaining hexokinase activity in a triple *∆hxk1*, *∆hxk2*, ∆*glk1* deletion strain [35].

**Fig. 2:**
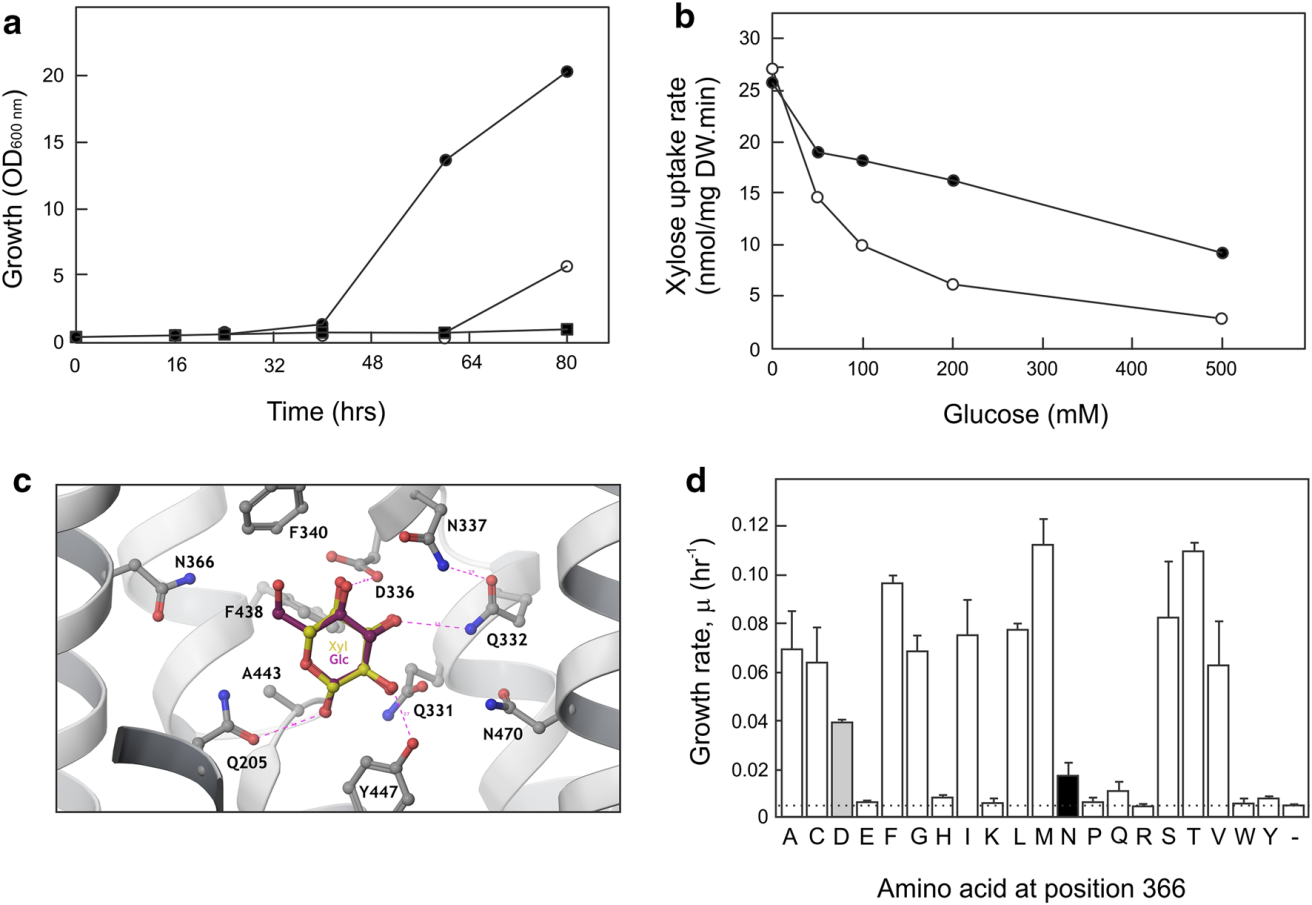
Characterization of the N366X mutants of *HXT11*. **a** Growth of the hexokinase deficient DS71054 strain containing the empty vector (*filled square*), or expression vectors bearing *HXT11* (*open circle*), and *HXT11*-N366D (*filled circle*) on 1 % xylose and 15 % glucose. **b** Uptake of [^14^C-] xylose by the DS68625 strain expressing *HXT11* (*open circle*) and *HXT11*-N366D (*filled circle*) in the presence of increasing concentrations of glucose. **c** Detailed view of the sugar-binding pocket of the Hxt11 homology model, showing the first shell amino acid side chains that interact with bound glucose (*cyan*) and xylose (*yellow*). N366 is located to the *left*, pointing the side chain towards the 6-OH and 6-CH_2_ of glucose. Most residues in this pocket are strictly conserved between Hxt11 and XylE, apart from D337 (I in XylE), A442 (G in XylE), Y446 (W in XylE) and N469 (Q in XylE). The scheme was constructed using Maestro (Schrödinger LLC, NY, USA). **d** Exponential growth rates (μ) of strain DS71054 expressing the indicated Hxt11-N366X mutants when grown on 10 % glucose and 1 % xylose. The *black bar* indicated the wild-type position, and the *gray bar* indicates the N366D mutant obtained in the error-prone mutagenesis. The *dashed line* indicates the growth rate of strain DS71054 without any introduced transporter. The *error bars* are the standard error of the mean from two technical samples

A library of mutagenized *HXT11* genes was cloned into the yeast vector pRS313-T7P7 and transformed into strain DS71054. Three thousand clones were screened for growth on xylose (1 %) in the presence of a 15-fold excess of glucose using a 96-well plate format. This screen yielded eight mutants that showed faster growth than the strain containing the wild-type Hxt11 transporter. The corresponding plasmids were isolated and re-sequenced. All eight plasmids contained the *HXT11* gene with the same mutation leading to a change at position 366 from an asparagine (N) into an aspartate (D) residue. When the Hxt11-N366D mutant was expressed in strain DS71054, it also displayed faster growth on 1 % xylose in the presence of a 15-fold excess of glucose compared to wild-type Hxt11 (Fig. 2a). When expressed in strain DS68625, Hxt11 and Hxt11-N366D showed a similar growth rate on xylose (Additional file 1: Figure S4), indicating that the mutation primarily affects the ability of glucose to compete with xylose transport, rather than improving xylose transport per se. Indeed, the affinity for glucose uptake by Hxt11-N366D was reduced 2.8-fold compared to the wild-type Hxt11, while there was only a negligible effect of the mutation on the xylose uptake affinity (Table 2). The *V*_max_ for glucose uptake by Hxt11-N366D was slightly increased compared to Hxt11, while the *V*_max_ for xylose uptake remained unchanged (Table 2). Because of these altered kinetic parameters, xylose uptake by the Hxt11-N366D mutant was notably less sensitive to glucose inhibition as compared to the wild-type Hxt11 (Fig. 2b).

To understand the molecular mechanism of the reduced affinity for d-glucose by the N366D mutation in Hxt11, a homology model for Hxt11 was constructed based on the crystal structure of XylE from *E. coli* with xylose bound in the occluded state (PDB ID: 4GBY) [37]. Notably, N366 is predicted to be located in a conserved region in transmembrane domain (TMD) 8 between Hxt11 and XylE, which has the sequence 363-GVV**N**-366 in Hxt11 and 322-GVI**N**-325 in XylE, where the conserved asparagine residue is marked in bold (Fig. 2c). Therefore, like N325 in the crystal structure of XylE, N366 in Hxt11 is in close proximity to the 5-CH_2_ of the ring form of d-xylose. In the case of a bound d-glucose in Hxt11 (Fig. 2c), the 6-CH_2_ and 6-OH would point towards N366, which upon mutation to an aspartate apparently lowers the affinity for d-glucose, while leaving d-xylose binding undisturbed. It is unclear why such a subtle change in the charge and not size of the amino acid side chain results in such a large effect on the affinity for d-glucose, especially in view of the lack of a direct interaction between the xylose 5-CH_2_ and modeled glucose 6-CH_2_ and 6-OH in XylE and the Hxt11 model. The answer may lie in the interactions between the sugar and amino acids in the outward open or inward open state of the transporter, for which co-crystal structures are as of yet unavailable.

To examine the mutational space, N366 was also replaced with each of the other 18 remaining amino acids to generate a series of N366X mutants. The corresponding genes were expressed in the hexokinase deficient strain DS71054 and evaluated for their ability to utilize 1 % xylose in the presence of 10 % glucose using 96-well plates. Growth on xylose in the presence of glucose was improved when N366 was substituted by a methionine (M) or threonine (T) residue (Fig. 2d), and was up to threefold improved compared to the N366D substitution. Amino acids with a positively charged (K, R), polar (Q, H) or bulky hydrophobic side chains (W, Y except for F) did not support growth on xylose under the screening condition, whereas in particular aliphatic side chains (L, I, V, M) or polar (S, T) supported enhanced growth on xylose in the presence of glucose. The mutants were also tested for growth on glucose when expressed in strain DS68625. The N366M and N366T Hxt11 mutants showed similar growth on glucose or on xylose as compared to the wild-type Hxt11 (Additional file 1: Figure S4). In addition, the expression level of all individual mutants was determined using GFP-tagged Hxt11 proteins (Additional file 1: Figure S5). All Hxt11-GFP proteins were highly expressed on the plasma membrane, except for the N366W and N366Y mutant Hxt11 proteins that showed a cytosolic and likely vacuolar localization of the fluorescence. This suggests protein misfolding and/or faulty targeting of these mutants and explains the low activity in the growth experiments with xylose and glucose.

Compared to the wild-type Hxt11, the affinity for glucose transport by the N366T and N366M Hxt11 mutants was reduced up to five- and fourfold, respectively (Table 2). In contrast, the affinity for xylose uptake by these mutants increased up to twofold relative to Hxt11 causing an overall tenfold improvement of the transporters specificity for xylose versus glucose. Strikingly, these mutant transporters exhibit a higher affinity for xylose transport compared to glucose, while the *V*_max_ for xylose uptake remained largely unchanged relative to Hxt11. These data demonstrate that the N366T and N366M mutations convert Hxt11 into a transporter with a greater specificity for xylose than for glucose.

All hexose transporters in *S. cerevisiae* including Hxt11m belong to the major facilitator superfamily (MFS) [42]. They share a high identity in the transmembrane region but differ substantially in size and amino acid sequence of their NH2- and COOH-terminal region. It was previously reported that in a chimeric transporter of Hxt1 and Hxt2, N331 in TMD 7 is a key residue responsible for the moderately high-affinity glucose transport activity of Hxt2 [43]. In addition, N340 of Hxt7, which corresponds to N331 of Hxt2 is a key residue that determines the glucose affinity in Hxt7, suggesting that the mechanism of substrate recognition is similar in Hxt7, Hxt2 and Hxt1 [44]. Also, the Gal2-N376V and Hxt7-N370S mutants that localize to TMD 8 were shown to be equipped with an improved affinity for d-xylose [28]. The latter residue corresponds the residue in Hxt11 as reported in this study lending further support for the notion that TM8 is crucial for substrate specificity.

### Co-fermentation of glucose and xylose by the mutant HXT11-expressing strain DS68625

**Fig. 3:**
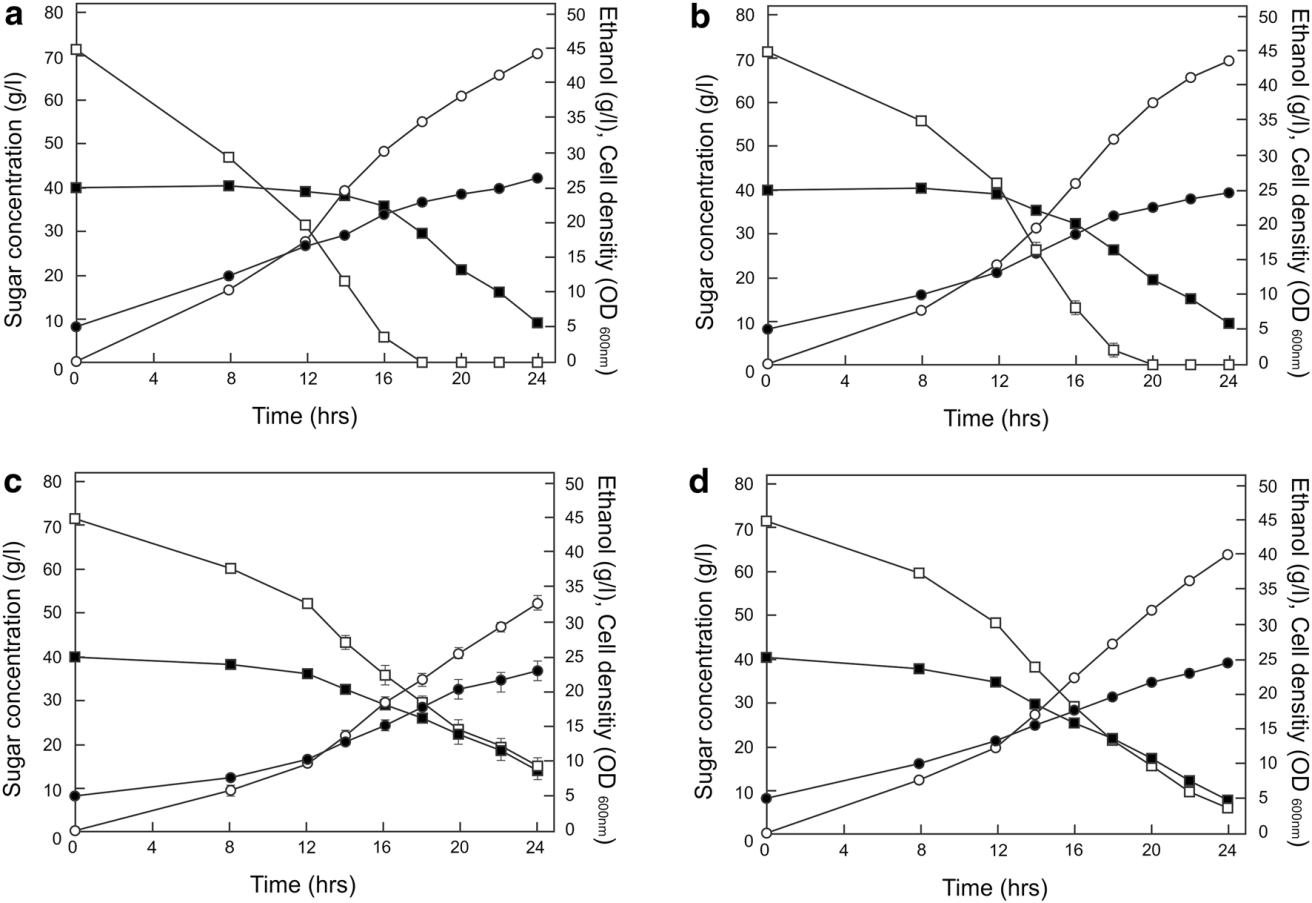
Consumption of xylose and glucose by the control strain DS68616 containing the empty vector expressing all endogenous Hxt transporters **a**, and the transporter-deficient strain DS68625 expressing Hxt11 **b**, Hxt11-N366M **c** or Hxt11-N366T **d**, respectively. *Symbols*: glucose (*open square*), xylose (*filled square*), ethanol (*open circle*), and cell density (*filled circle*). The xylose and glucose concentration was 4 and 7 %. The *error bars* are the standard error of the mean from three independent experiments with triple samples

Because of the marked effects of the N366 mutations in Hxt11 on the sugar specificity, mutants were further examined for their ability to co-metabolize xylose and glucose under industrial relevant sugar concentrations, i.e., 7 % glucose and 4 % xylose under anaerobic conditions which is experimentally an usually stringent condition for co-metabolism validation as other studies use equimolar concentrations. N366M Hxt11 (Fig. 3c) and in particular N366T Hxt11 (Fig. 3d) supported a near to perfect co-consumption of glucose and xylose when expressed in strain DS68625, except for the early stages of fermentation at the high glucose concentrations where glucose metabolism was slightly preferred. Effective co-consumption was also observed when the mutants were grown at equal glucose and xylose concentrations, i.e., 1.0 % each (Additional file 1: Figure S6). Compared to the the wild-type Hxt11 (Fig. 3b) and the DS68616 strain containing the full complement of hexose transporters (Fig. 3a), co-consumption is due to reduced and increased rates of glucose and xylose conversion, respectively. Importantly, the wild-type Hxt11 (Fig. 3b) and the DS68616 strain (Fig. 3a) showed a severely delayed consumption of xylose which is typically observed in co-metabolism events.

For further analysis, the specific xylose consumption rates were measured during the anaerobic cultivation performed in the presence of 7 % glucose. The specific xylose consumption rate of strain DS68625 with Hxt11 N366T was improved to 0.63 g/g cell h compared to 0.49 and 0.54 g/g cell h for strain DS68616 and strain DS68625 expressing Hxt11p, respectively. On the other hand, the specific glucose consumption rate with Hxt11 N366T was reduced to 1.23 g/g cell h compared to 1.74 g/g cell h for Hxt11p (Additional file 2: Table S2). Additionally, the xylose consumption rate in the presence of various concentrations of glucose was improved up to twofold for strain DS68625 carrying the Hxt11 N366T and N366M mutants as compared to the same strain with Hxt11 or strain DS68616 (Additional file 1: Figure S7). The ethanol productivity of these improved DS68625 strains was only slightly lower compared to strain DS68616 but the ethanol yield was the same (Additional file 2: Table S2). Overall, these data demonstrate that the mutagenesis of the N366 position of Hxt11 yields mutants that as a single transporter mediates a balanced uptake of glucose and xylose, thereby supporting co-consumption of the these sugars at overall sugar conversion rates that are in line with the rates observed in a strain using the full complement of native transporters.

Although the previously described Gal2-N376V and Hxt7-N370S mutants that localize with an improved affinity for d-xylose [28] localize in the same helical region, i.e., TMD 8, as with Hxt11 of this in this report, no co-consumption of glucose and xylose could be shown. This is likely because of the very low xylose transport rates by the mutant Hxt7 and Gal2 transporters compared to the Hxt11 transporter identified in this study. Also, the recently described Hxt7-N370S mutant still showed a higher affinity for glucose compared to xylose [28]. Recently, we also obtained mutants of Hxt36 with an improved d-xylose affinity [41]. In particular, the mutant Hxt36-N367A supported d-glucose and d-xylose co-consumption when both sugars are present in equimolar amounts. Also, the heterologous expression of xylose transporters Gxf1 from *Candida intermedia*, Sut1 from *Pichia stipitis and* At5g59250 from *Arabidopsis thaliana* was reported to improve xylose utilization by a recombinant *S. cerevisiae* [12, 16, 38]. For the Gxf1 transporter, the improvement of xylose utilization was restricted to substrate concentrations >10 g/L [12], whereas the Sut1 and At5g59250 transporters increased xylose uptake at substrate concentrations >50 g/L [16]. In addition, xylose-utilizing *S. cerevisiae* was not capable of producing ethanol at a low concentration of xylose (<10 g/L), although the strain consumed xylose. During co-fermentation of xylose and glucose, the consumption rate of xylose is low as long as there is still glucose present in the growth medium [39]. Therefore, the present study demonstrates that Hxt11 is a more efficient transporter for ethanol production on sugar mixtures containing glucose and xylose as compared to the heterologously expressed specific xylose transporters mentioned above.

## Conclusions

Here, we report for the first time that expression of the cryptic Hxt11 sugar transporter of *S. cerevisiae* stimulates xylose utilization both under aerobic and anaerobic conditions. Hxt11 has previously been overlooked as it is normally not expressed in *S. cerevisiae.* Xylose transport via Hxt11 is, however, less sensitive to glucose inhibition as compared to transport via the main hexose transporters in yeast such as Hxt2. The selectivity could further be enhanced by direct screening of Hxt11 mutants at position N366 for glucose-insensitive transport of xylose. When expressed in a sugar transporter-deficient strain of *S. cerevisiae*, selective mutants supported the co-consumption of xylose and glucose in mixed sugar fermentations using industrially relevant sugar concentrations. Because of the single transporter solution for co-consumption, future studies should be directed at increasing the rates of transport and metabolism to also reduce the duration of the fermentation. Our results demonstrate that endogenous transporter engineering is a powerful approach yielding a solution to a critical step that limits the use of yeast for the industrial fermentation of lignocellulosic biomass.

## Methods

### Yeast strains and chemostat culture conditions

*S. cerevisiae* strains used in this study (Table 1) were provided by DSM and may be made available for academic research under a strict Material Transfer Agreement with DSM (contact: paul.waal-de@dsm.com). The culture medium used was a defined mineral medium, with vitamins prepared as described by Verduyn et al. [40]. For storage of the strains, shake flask cultures were performed in the mineral medium supplemented with 2 % maltose (Sigma-Aldrich) in case of the DS68616/DS68625/DS68625-evo-derivatives, or 2 % xylose (Roth) in case of DS71054-derivatives. With strains DS68625, DS68616, or DS71054, the Verduyn-urea was supplemented with 0.02 % histidine (Sigma-Aldrich) to complement for the auxotrophic marker. Cultures were maintained at 30 °C in an orbital shaker until stationary growth phase was reached. After the addition of glycerol to 30 % (v/v), samples were stored in 2 ml aliquots at −80 °C.

Chemostat cultures were grown in a 3-L stirred tank bioreactor (Applikon, Schiedam, The Netherlands) filled with 500 ml of the mineral medium at a temperature of 30 °C, pH 4.5 and at a dilution rate of 0.01–0.05 h^−1^. Stirring was performed at 400 rpm and the starting OD_600_ was 1. Some diffusion of oxygen was allowed via the inflowing medium and the venting vessel on the reactor.

### Hexose transporter deletion strain

The eight major HXT (*HXT1*–*7* and *GAL2* [21]) genes were deleted to generate a xylose transport-negative model strain for screening. *S. cerevisiae* strain DS68616 equipped with DSM proprietary xylose fermentation technology based on Kuyper et al. [5, 7] was used for the following genetic modifications. Genes were deleted by standardly disrupting the target locus by integrating a floxed dominant resistance marker; markers were surrounded by *lox*P sites to allow for CRE-meditated marker removal and recycling [41]. Locus-specific flanks were 400–700 bp large and PCR-amplified using the primers listed in Additional file 2: Table S3. Up- and downstream flanks were fused to the floxed dominant resistance markers by standard cloning methods, and re-amplified using the forward primer of the upstream flank (HXT*x*uf) and the reverse primer of the downstream flank (HXT*x*dr).

First, the *HIS3* gene was deleted to serve as a marker for the introduction of putative enhanced xylose transporter(s) (libraries) as described below. The histidine auxotroph DS68616 was generated by the insertion of *lox*P-*kan*MX-*lox*P at the *HIS3* locus. Subsequently, the marker was removed by CRE-mediated marker removal [41]. For the deletion of the eight main hexose transporter genes (*HXT1*–*7* and *GAL2*) in *S. cerevisiae*, four deletion constructs were generated bearing either the *kan*MX, *zeo*MX, *hph*MX, *nat*MX [42, 43]. The hexose transporters were deleted in the following order: (1) *HXT3*-*HXT6*-*HXT7* cluster, (2) *HXT5*-*HXT1*-*HXT4* cluster, (3) *GAL2*, (4) *HXT2*. All transformations were plated on yeast extract (10 g L^−1^), peptone (20 g L^−1^) agar (15 g L^−1^) medium supplemented with 20 g L^−1^ maltose. Maltose was added to the medium, because the uptake of this disaccharide occurs via an alternative transport system other than the Hxt glucose transporters [26]. With each deletion of a (cluster of) *HXT* gene(s), an additional marker was inserted in the order: (1) *hph*MX, (2) *nat*MX, (3) *zeo*MX, (4) *kan*MX. With each inserted additional marker, the respective antibiotic was additionally supplemented to the medium in the following order: (1) HG, (2) HG and nour, (3) HG, nour and phleo, (4) HG, nour, phleo and G418. After integration of all four deletion constructs, a single colony was isolated under selection of all four antibiotics. The resulting strain, DS68625, did not grow on mineral medium (Verduyn-urea; based on Luttik et al. [44]) with 2 % xylose, and showed a delayed growth pattern on Verduyn-urea with 2 % glucose (data not shown). Correct integrations were verified by PCR analysis on genomic DNA isolates.

### Construction of hexokinase deletion strain

A hexokinase deletion strain was constructed to allow screening for variants with improved ability to transport xylose in the competitive presence of glucose. Previously, a triple hexokinase mutant has been described to investigate the specificity for xylose transport (*Δhxk1*, *Δhxk2*, *Δglk1*; [45]). However, the additional deletion of galactokinase gene *GAL1* is necessary since Gal1p can easily acquire SNPs enabling glucose conversion [35]. To prevent this, *GAL1* was deleted. For deletion of hexokinase genes, oligonucleotides were designed (listed in Additional file 2: Table S4; numbers 834, 835, 838, 839, 846, 847, 848, 849) comprised of 60 nucleotide flanking sequences homologous to the hexokinase gene locus, and of 20 nucleotides homologous to a floxed dominant resistance marker cassette (similar as described above except that *kan*MX was surrounded by *lox*66 and *lox*71). The oligonucleotides were used to amplify the deletion constructs. Subsequent PCR products were column filter-purified (GeneJet Kit, Thermo Fischer, Landsmeer, The Netherlands) and used for transformations experiments.

For the generation of a strain incapable of hexose metabolism but capable of hexose transport, four hexokinase gene deletions were made in the xylose-fermenting strain DS71055 (Table 1). Similar as in the DS68625 lineage, HIS3 was deleted to use as marker for the introduction of transporter constructs, similar as described for the other screening assay. The hexokinase and marker genes were deleted in the following order: (1) *GLK1*, (2) *HXK1*, (3) *GAL1*, (4) *HXK2*, (5) *HIS3* integrating floxed *kan*MX, *nat*MX (followed by round of marker recycle), *hph*MX, *kan*MX, and *nat*MX (followed by round of marker recycle), respectively. After the deletion of *GLK1* and *HXK1* both the integrated *kan*MX and *nat*MX marker were recycled by galactose-induced Cre-mediated recombination. After deletion of *HXK2,* the intermediate strain was maintained on xylose-containing rich medium (YPX). After *HIS3* deletion the integrated *hph*MX and *kan*MX markers were removed by galactose-induced CRE recombination. To ensure growth of the strain, 2 % xylose was added to YP 2 % galactose + nourseothricin (YPGX). Selection on nourseothricin ensured maintenance of the *nat*MX marker at the *HIS3* locus leaving a selection trait to be used possibly later on. After single colony isolation, the strain was verified for its deletions and by colony PCR, and designated as DS71054. The final quadruple hexokinase mutant DS71054 was screened for growth on Verduyn-urea-his supplemented with 2 % glucose or xylose. DS71054 did not grow on glucose, but did grow on xylose (Additional file 1: Figure S3). DS71054 was further maintained on YPX for storage and handling.

### Genomic DNA isolation, RNA extraction and cDNA synthesis

Genomic DNA was isolated after 16 h of growth of the cells in the mineral medium containing 2 % maltose using a modified yeast genomic DNA isolation protocol [46]. Yeast pellets from 2 ml of exponential phase cell culture were mixed with 0.2 ml of glass beads (diameter, 0.45 mm) and disrupted in a Fastprep FP120 (Bio-101, Thermo Savant, California, USA) by a 45-s burst at speed 6.

Total RNA was isolated from the parental DS68625 and evolved DS68625-evo strains by a glass-bead disruption/Trizol extraction procedure. Yeast pellets from 2 ml of exponential phase cell culture were mixed with 0.2 ml of glass beads (diameter, 0.45 mm) and 0.9 ml of Trizol with 125 μl chloroform, and disrupted in a Fastprep FP120 (Bio-101, Thermo Savant, California, USA) by a 45-s burst at speed 6. Extracted total RNA (1 μg) was converted into cDNA using the iScript Kit (Bio-Rad, US).

### Real-time PCR

*HXT8*–*HXT17* specific real-time PCR was performed using the SensiMix SYBR and Fluorescein kit (Quantace Ltd) and a MyiQ iCYCLER Real Time PCR instrument (Bio-Rad, Veenendaal, The Netherlands). Each 25-μl reaction contained 12.5 μl of SYBR green Master Mix, 4 μl of extracted DNA, 0.5 μl of each primer (10 nM) and 7.5 μl of sterile water. The PCR condition for HXT8-16 were 10 min at 95 °C followed by 39 cycles of amplification (15 s at 95 °C, 30 s at 60 °C, 30 s at 72 °C). The primers shown in Additional file 2: Table S5 were utilized to amplify the mRNA fragments by PCR.

### Overexpression *HXT11* and *HXT12* in *S. cerevisiae*

For PCR amplification, Phusion^®^ High-Fidelity PCR Master Mix with HF buffer was used (Finnzymes; Fisher Scientific, Landsmeer, The Netherlands). Restriction enzymes and T4 DNA ligase were acquired from Fermentas (Fisher Scientific, Landsmeer, The Netherlands). The open-reading frames for *HXT11* and *HXT12* were PCR-amplified from cDNA of the wild-type DS68625 using primers HXT11F (5′-GGCCTCTAGAATGTCAGGTGTTAATAATACATCCGC-3′), HXT12F (5′-GGCCTCTAGAATGGGTTTGATTGTCTCAATATTCAAC-3′) and HXT11•12R (5′-CGATGGATCCTCAG CTGGAAAAGA ACCTCTTGTA AATTG-3′). The HXT11 and HXT12 PCR fragments were sequenced for validation, cut using restriction enzymes *Xba*I and *Bam*HI (Fermentas) and cloned in yeast expression vector pRS313-P7T7 behind the constitutive HXT7 (−391) promoter (P7) preceding the HXT7 terminator (T7). Plasmids were amplified and maintained in *E. coli* DH5α cells. Plasmids were isolated from *E. coli* cultures using the GeneElute plasmid Miniprep kit (Sigma-Aldrich, The Netherlands). The *HXT11* and *HXT12* expression construct were transformed into *S. cerevisiae* DS68625 using standard yeast genetic techniques [47].

### Construction of *HXT11*-*GFP* expression plasmids

The GFP gene in plasmid pDONR-eGFP-AT [48] was amplified by PCR with the primers GFP-F and GFP-R, and the product was cloned into the *Xma*I and *Cla*I restriction sites of pRS313-P7T7. The resulting plasmid and PCR products of the N366X mutants of *HXT11* were digested with *Xba*I and *Xma*I, and ligated with the GFP cassette. *HXT11*-*GFP* chimeras were validated by DNA sequencing.

### Mutagenesis and screening for xylose transport mutants of Hxt11

Error-prone PCR experiments were performed following the standard protocol [49] using DNA Taq polymerase (Thermo) with 10 ng of template DNA in 100 μl of PCR mix containing 5.5 mM MgCl_2_ and 0.15 mM MnCl_2_. The library of fragments was cloned into pRS313-P7T7 vector and transformed into the strain DS71054 that is unable to grow on glucose but that contains the xylose metabolic pathway. Three thousand variants were screened by growing the transformants on a 1:15 ratio of xylose (1 %) and glucose (15 %) with mineral medium using the 96-well plate format and Synergy MX (BioTek Instruments, Inc, USA) reader.

Saturated mutagenesis of position N366 in Hxt11 was done by PCR using Phusion^®^ High-Fidelity PCR Master Mix with HF buffer using primer pairs F HXT11 *Xba*I/R HXT11 366NNN and F HXT11 366NNN/R HXT11 *Bam*HI (Additional file 2: Table S6). The fragments of 1113 and 591 base pairs were subsequently used in an overlap PCR using the outside primers F HXT11 *Xba*I and R HXT11 *Bam*HI and cloned into pRS313P7T7 using *Xba*I and *Bam*HI. Sequencing of 48 *E. coli* clones yielded N367S (tct), N367P (cca), N367G (ggt), N367A (gcc), N367H (cac), N367R (cgc), N367L (ttg), N367C (tgt), N367T (acg), N367D (gat), N367Q (caa), and N367V (gtg). The remaining six amino acids at position 366 were amplified and cloned as mentioned above with overlap PCR using specific primers in which the NNN was replaced by ttt (F), gag (E), tgg (W), atg (M), aaa (K), and N367Y (tat).

### Deletion of the HXT11 gene and selection of knockout HXT11 strain

For construction of the deletion plasmid pRS313-P7T7-HIS (carrying *loxP*-*his*-*loxP*), the *S. cerevisiaehis* gene including *HXT11* promoter and terminator was amplified with oligonucleotides KOP11 (5′-AATAATCATTGCACAATTTAGTTCTAAACGCTTTTGTTATTACTCAATATCCGTTTTAAGAGCTTGGTGAGCGCTAGGAGTC-3′) and KOT11 (5′-TCGTCAATTTTTTTTTTTGCTTTTTTACCAATTTACCGAAAACTAGAAGAGAGTTCAAGAGAAAAAAAAAGAAAAAGCAAAAAGAAAAAAGGAAAGCGCGC-3′) (Histidine gene underlined). The PCR fragment was transformed into the strain DS68625-evo and transformants were initially screen on 2 % maltose plate for growth. Subsequently, to test growth differences of the *HXT11* deletion in the DS68625-evo strain, cells were grown on 2 % xylose for 72 h at 30 °C, diluted to OD600 0.1 and grown to OD600 1.5–2 in 96-well plates. Genomic DNA was isolated from the yeast cells that were not able to grow on 2 % xylose. To verify the deletion, the *HXT11* gene was amplified with oligonucleotides delta-HXT11F (5′-ATGTCAGGTGTTAATAATACATCCGC-3′) and delta-HXT11R (5′-TCAGCTGGAAAAGAACCTCTTGTAAATTG-3′), to yield a PCR fragment of 1704 bp. The PCR cycle was as follows: 35 cycles of denaturation at 98 °C for 10 s, annealing at 65 °C for 10 s and extension at 72 °C for 60 s followed by final extension for 10 min at 72 °C.

### Uptake measurements

The uptake of radiolabeled xylose or glucose was measured as follows: cells were collected by centrifugation (3000 rpm, 3 min, 20 °C), washed and suspended into mineral medium. [^14^C] xylose or [^14^C] glucose (CAMPRO scientific GmbH, Veenendaal, The Netherlands) stocks were added to the cells, uptake reactions were stopped at various time intervals by addition of 5 ml of 0.1 M lithium chloride, and the suspension was filtered (0.45 μm HV membrane filter, Milipore, France). Filters were washed with another 5 ml of lithium chloride and counted with the emulsifier scintillator plus (Perkin-Elmer, USA). Uptake experiments with strain DS68625 expressing Hxt11-variants were done with 0.5–500 mM xylose or 0.1–500 mM glucose. In general, the uptake of xylose was monitored after 1 min and for glucose after 15 s in quadruple. Glucose competition studies were performed with 50 mM [^14^C] xylose in the presence of 50–500 mM unlabeled glucose.

### Molecular modeling

The molecular modeling software package, including Prime 3.1 and Maestro 9.3, from Schrodinger LLC (NY, USA) was used to build a homology model for Hxt11. The crystal structure of XylE from *E. coli* with xylose bound (PDB ID: 4GBY) [37], which is 31.6 % identical to Hxt11, was used as a template for the model building. Because of the lower identity, the sequence alignment between Hxt11 and XylE was manually corrected using information from family sequence alignments in the program Prime to accurately reflect the correct location of amino acid insertions and deletions.

### Fermentation

Yeast cultures were grown in mineral medium containing 2 % maltose for prepare inoculums for glucose and xylose fermentation experiments. Cells at mid-exponential phase were harvested and inoculated after washing twice by sterilized water. Fermentation experiments were performed using 100 ml of mineral medium containing 7 % glucose and 4 % xylose in 120 ml bottle at 30 °C with initial OD_600_ of 5 under oxygen-limited conditions. All of the bottle fermentation experiments were repeated independently.

### Fluorescence microscopy

Plasmid pRS313-P7T7:*GFP*-*HXT11* was transformed into *S. cerevisiae* strain DS68625. Individual colonies were used to inoculate minimal medium containing 2 % maltose, and cells recovered from the exponential growth phase (at an optical density of 10 at 600 nm) were subjected to fluorescence microscopy using a Nikon Eclipse-Ti microscope equipped with a 100× oil immersion objective, a filter set for GFP, and a Nikon DS-5Mc cooled camera (Nikon, Japan). Routinely, at least 100 cells per sample were examined, and each experiment was replicated at least three times.

### Miscellaneous analytical methods

*S. cerevisiae* cells were grown in the mineral medium supplemented glucose and xylose with the initial pH adjusted to 4.5. Cell growth was monitored by optical density (OD) at 600 nm using UV–visible spectrophotometer. The concentration of glucose, xylose, and ethanol was measured by HPLC (Shimadzu, Kyoto, Japan) using an Aminex HPX-87H column (Bio-Rad, USA) and a refractive index detector (Shimadzu, Kyoto, Japan). The temperature of the column and detector was maintained at 65 °C. The mobile phase was 0.005 N H_2_SO_4_ at a flow rate of 0.55 ml/min.

### Genomic analysis

For genome sequencing (BaseClear Inc., Leiden, The Netherlands), genomic DNA of strain DS68625 and DS68625-evo was isolated to prepare for sequencing libraries using modified yeast genomic DNA isolation protocol [46]. The Illumina Miseq system was used with paired-end gDNA sequencing, and for analysis, the data were mapped to a reference genome (CEN.PK 113-7D) in Genebank. The results from the mapping were used to identify single-nucleotide polymorphism (SNPs) and insertions and deletions (INDELS) in the reads using the CLC Genomics Workbench (CLC bio).

## Additional files

10.1186/s13068-015-0360-6 Supplementary figures. **Figure S1.** Growth (○). glucose utilization (□) and ethanol production (●) by the control strain DS68616 expressing all endogenous Hxt transporters and containing the empty vector (A), and the transporter-deficient strain DS68625 expressing Hxt11 (B). **Figure S2.** Xylose uptake by strain DS68625 expressing HXT2 (●) or HXT11 (○) in the presence of increasing glucose concentrations (0-500 mM). **Figure S3.** Growth of strain DS68616 (■) and DS71054 (□) on 2 % glucose (A) or 2 % xylose (B). **Figure S4.** Maximum exponential growth rate of strain DS68625 expressing various Hxt11-N366X mutants on 2 % glucose (A) or 2 % xylose (B). The black bar indicated the wild-type position, and the gray bar indicates the N366D mutant obtained in the error-prone mutagenesis. The dashed line indicates the growth rate of strain DS68625 without any introduced transporter. The error bars are from two technical samples. **Figure S5.** Fluorescence images of strain DS68625 expressing GFP fusion proteins of Hxt11-N366X mutants grow on 2 % maltose. **Figure S6.** Consumption of xylose and glucose at 1.0 % each by the transporter-deficient strain DS68625 expressing Hxt11 (A) or Hxt11-N366T (B). Symbols: glucose (□), xylose (■), and ethanol (○). The error bars are from two technical samples. **Figure S7.** Ratio of the Xylose and glucose consumption rate in the presence of glucose. Strain DS68616 with the empty vector (●), or strain DS68625 with a vector expressing Hxt11(○), Hxt11 N366M (■) or Hxt11 N366T (□). Data calculated from the fermentation profiles presented in Fig. 3.
10.1186/s13068-015-0360-6 Supplementary tables. **Table S1**. SNPs identified in the DS68625-evo strain by genome sequencing. **Table S2.** Fermentation profiles by the selected S. cerevisiae strain expressing Hxt11 and the Hxt11 N366M and N366T mutants during anaerobic batch cultivation. **Table S3.** Oligonucleotides used in hexose transporter strain construction. **Table S4.** Oligonucleotides used for construction hexokinase deletion strain. **Table S5.** Oligonucleotides used in qPCR. **Table S6.** Primers used for saturation mutagenesis of HXT11.

## Abbreviations

GLK1: glucokinase
GAL1: galactokinase
HXK: hexokinase isozymes
HXT: hexose transporter
SNP: single-nucleotide polymorphism
SUMO: small ubiquitin-like modifier
TMD: transmembrane domain
PCR: polymerase chain reaction
XKS: xylulose kinase
