# Supplementary material for: An engineered cryptic Hxt11 sugar transporter facilitates glucose–xylose co-consumption in Saccharomyces cerevisiae
Source: Biotechnol Biofuels. 2015 Nov 2;8:176. doi: 10.1186/s13068-015-0360-6 (PMC4630928; doi:10.1186/s13068-015-0360-6)
Supplement: Supplementary file 1 — 10.1186/s13068-015-0360-6 Supplementary figures. Figure S1. Growth (○). glucose utilization (□) and ethanol production (●) by the control strain DS68616 expressing all endogenous Hxt transporters and containing the empty vector (A), and the transporter-deficient strain DS68625 expressing Hxt11 (B). Figure S2. Xylose uptake by strain DS68625 expressing HXT2 (●) or HXT11 (○) in the presence of increasing glucose concentrations (0-500 mM). Figure S3. Growth of strain DS68616 (■) and DS71054 (□) on 2 % glucose (A) or 2 % xylose (B). Figure S4. Maximum exponential growth rate of strain DS68625 expressing various Hxt11-N366X mutants on 2 % glucose (A) or 2 % xylose (B). The black bar indicated the wild-type position, and the gray bar indicates the N366D mutant obtained in the error-prone mutagenesis. The dashed line indicates the growth rate of strain DS68625 without any introduced transporter. The error bars are from two technical samples. Figure S5. Fluorescence images of strain DS68625 expressing GFP fusion proteins of Hxt11-N366X mutants grow on 2 % maltose. Figure S6. Consumption of xylose and glucose at 1.0 % each by the transporter-deficient strain DS68625 expressing Hxt11 (A) or Hxt11-N366T (B). Symbols: glucose (□), xylose (■), and ethanol (○). The error bars are from two technical samples. Figure S7. Ratio of the Xylose and glucose consumption rate in the presence of glucose. Strain DS68616 with the empty vector (●), or strain DS68625 with a vector expressing Hxt11(○), Hxt11 N366M (■) or Hxt11 N366T (□). Data calculated from the fermentation profiles presented in Fig. 3. [file 13068_2015_360_MOESM1_ESM.docx]

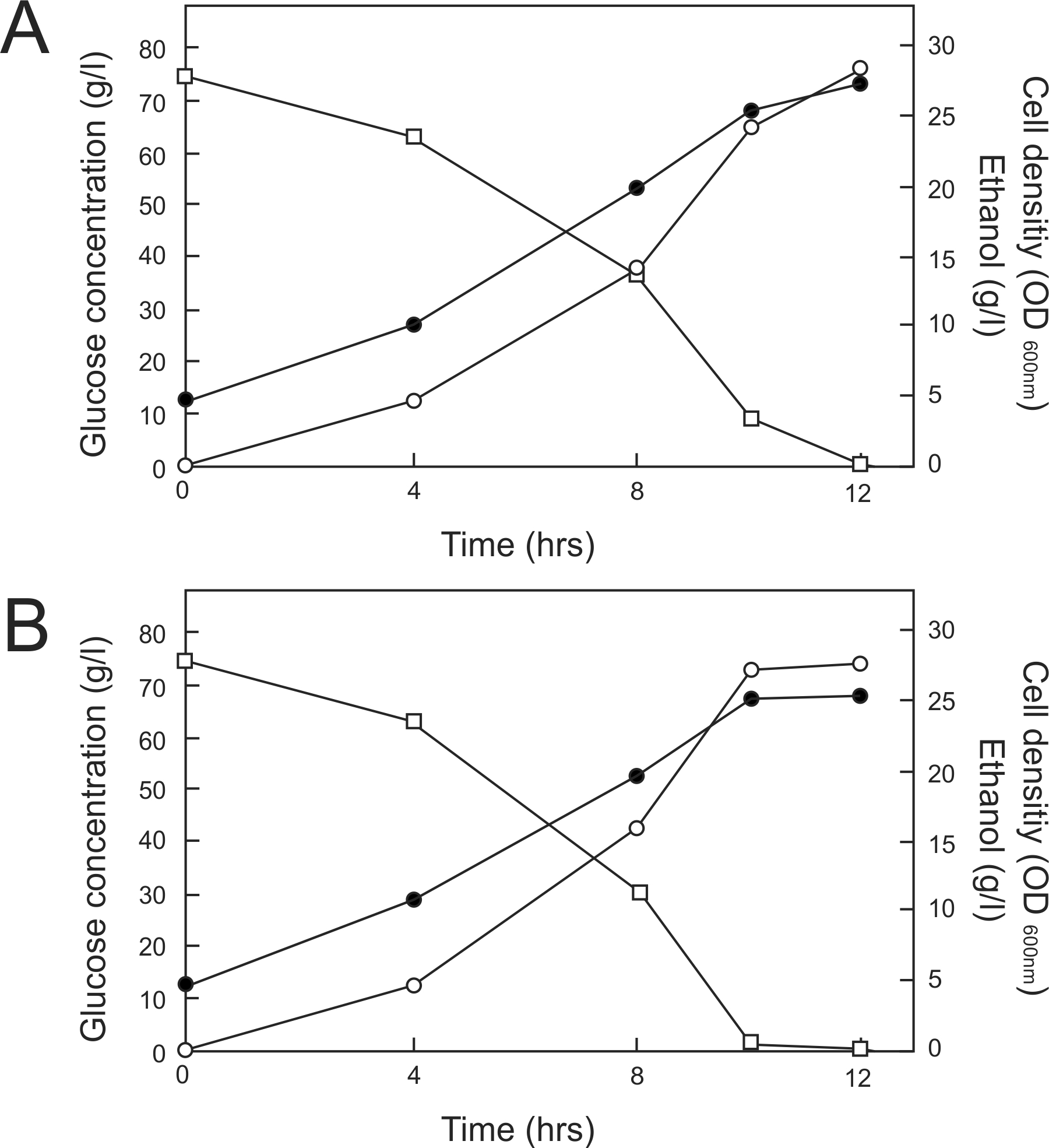


**Supplementary Fig. 1.** Growth (○). glucose utilization (□) and ethanol production (●) by the control strain DS68616 expressing all endogenous Hxt transporters and containing the empty vector (A), and the transporter-deficient strain DS68625 expressing Hxt11 (B).

**
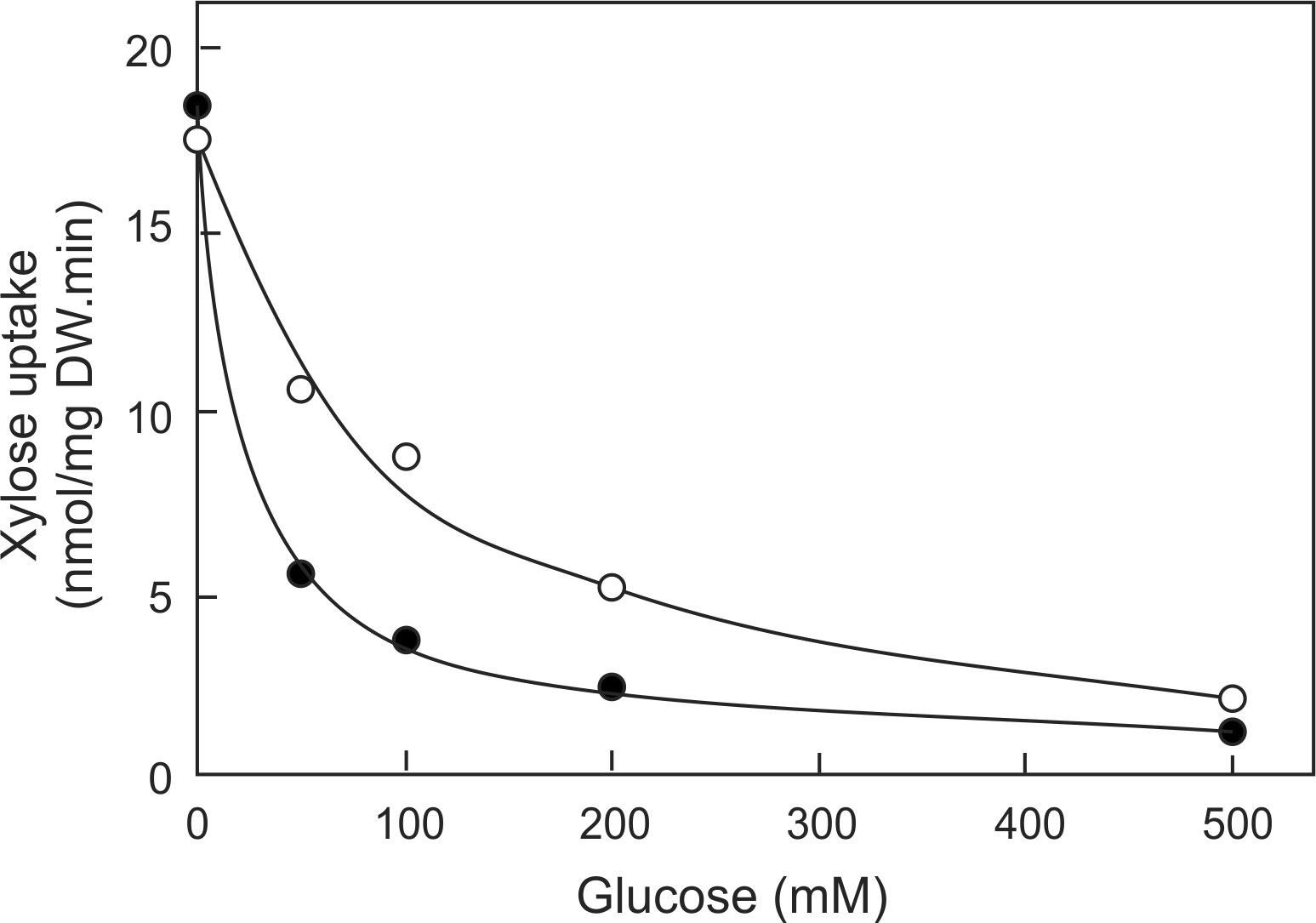
**

**Supplementary Fig. 2.** Xylose uptake by strain DS68625 expressing *HXT2* (●) or HXT11 (○) in the presence of increasing glucose concentrations (0-500 mM).


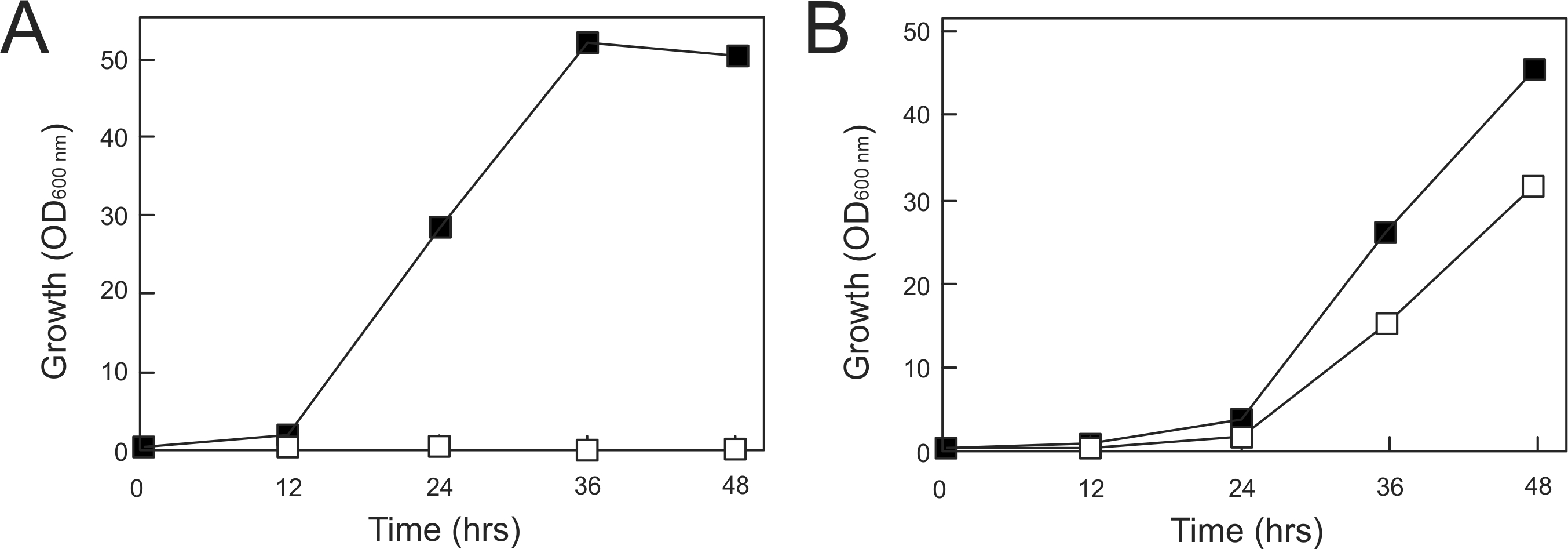


**Supplementary Fig. 3.** Growth of strain DS68616 (■) and DS71054 (□) on 2 % glucose (A) or 2 % xylose (B).


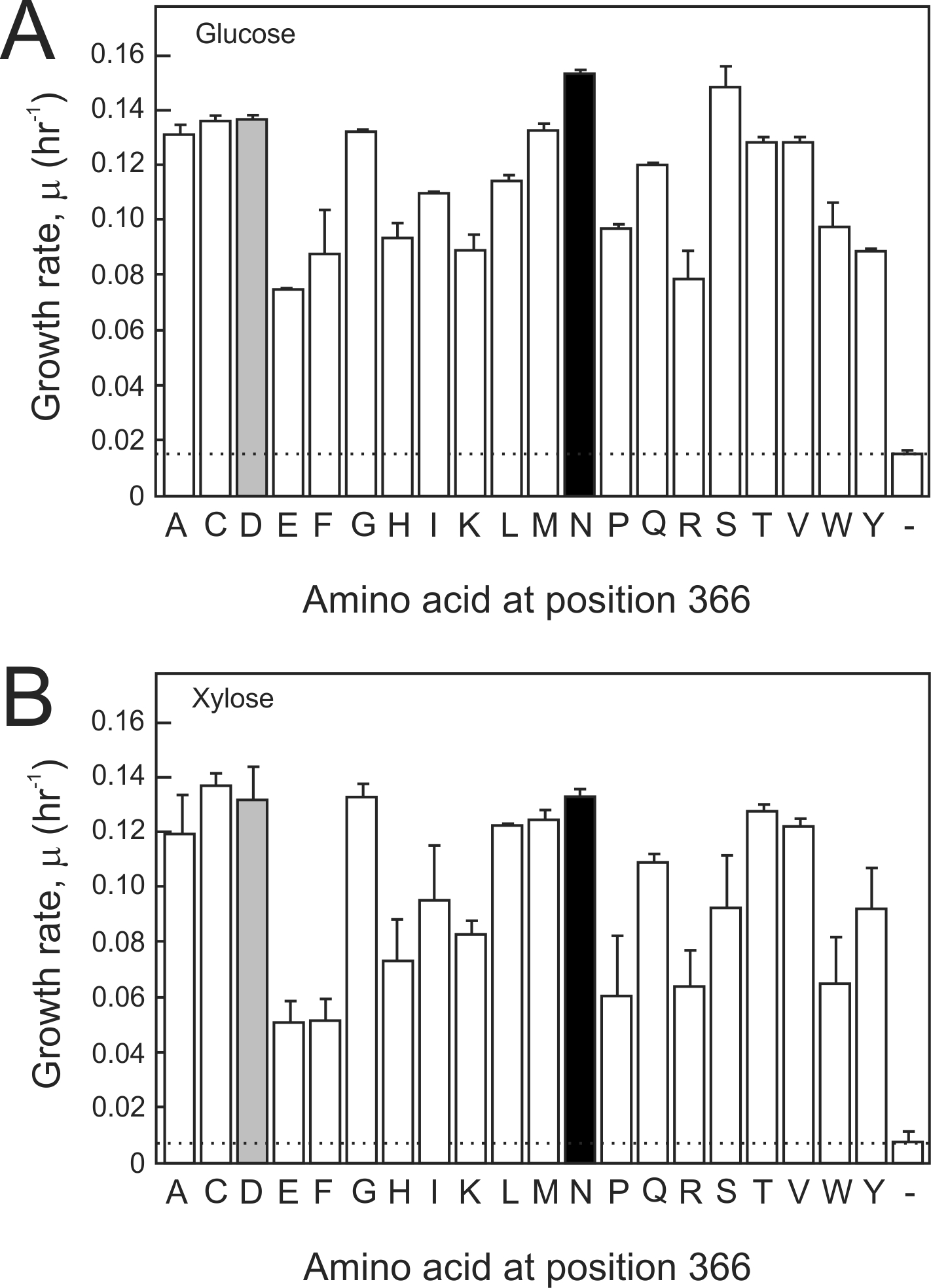


**Supplementary Fig. 4.** Maximum exponential growth rate of strain DS68625 expressing various Hxt11-N366X mutants on 2 % glucose (A) or 2 % xylose (B). The black bar indicated the wild-type position, and the gray bar indicates the N366D mutant obtained in the error-prone mutagenesis. The dashed line indicates the growth rate of strain DS68625 without any introduced transporter. The error bars are from two technical samples.


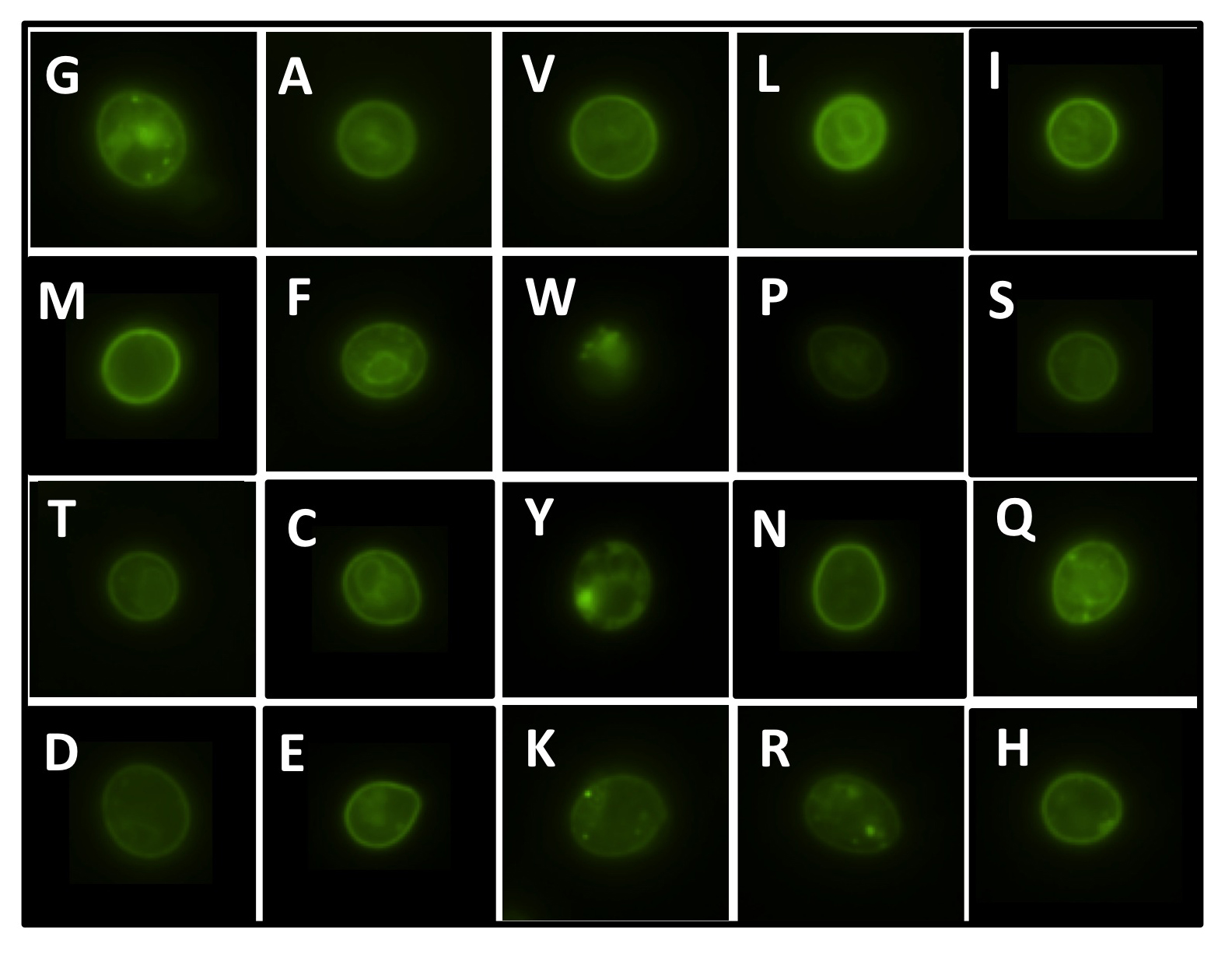


**Supplementary Fig. 5.** Fluorescence images of strain DS68625 expressing GFP fusion proteins of Hxt11-N366X mutants grow on 2 % maltose.


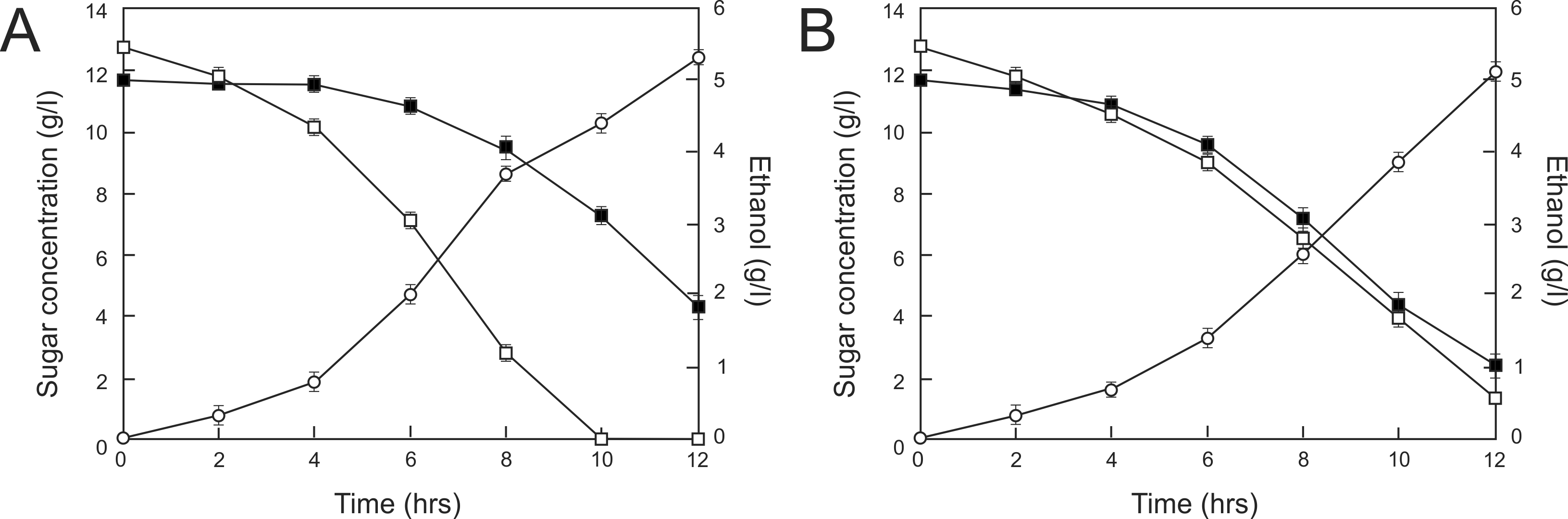


**Supplementary Fig. 6.** Consumption of xylose and glucose at 1.0 % each by the transporter-deficient strain DS68625 expressing Hxt11 (A) or Hxt11-N366T (B). Symbols: glucose (□), xylose (■), and ethanol (○). The error bars are from two technical samples.

**
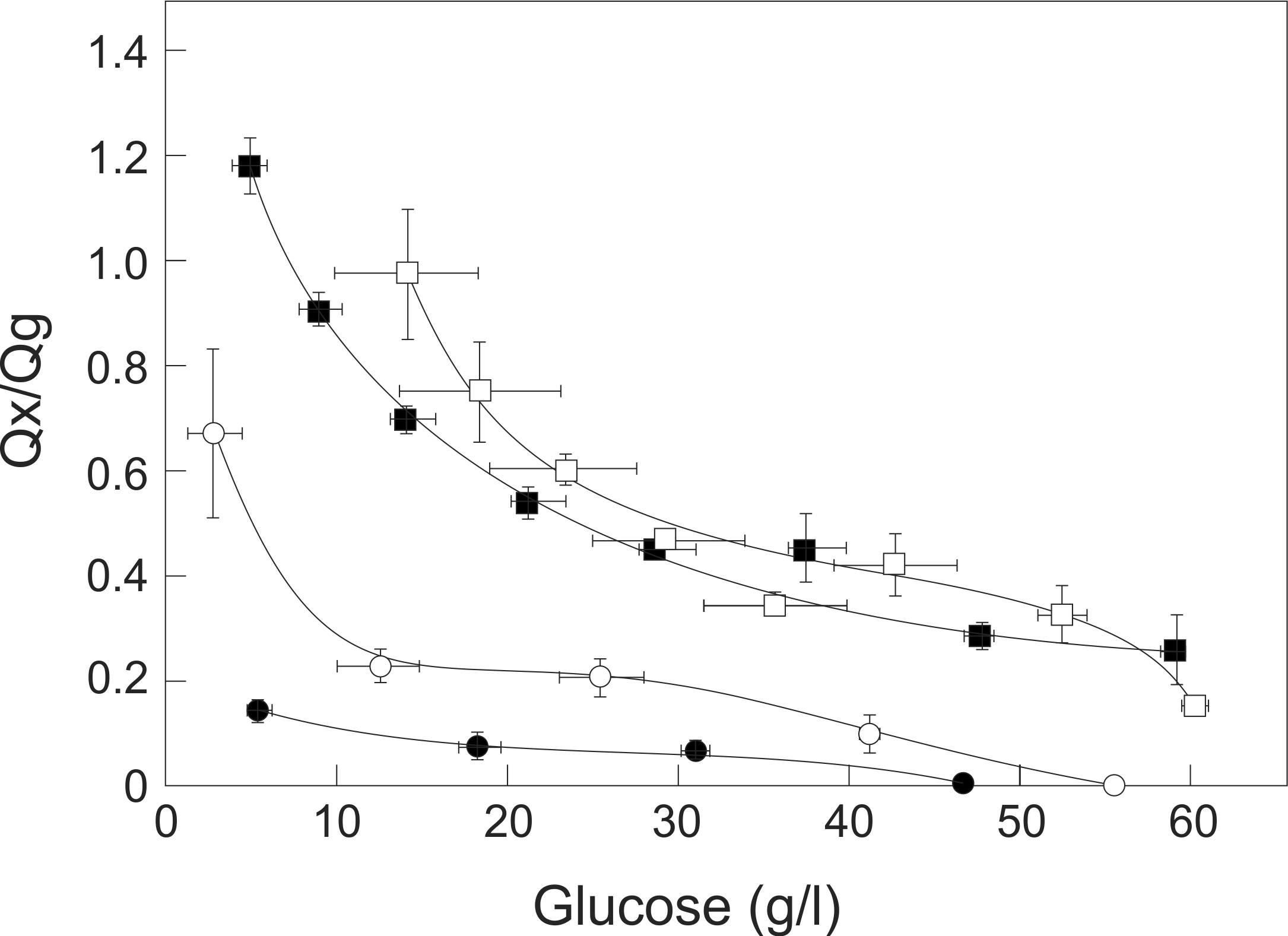
**

**Supplementary Fig. 7.** Ratio of the Xylose and glucose consumption rate in the presence of glucose. Strain DS68616 with the empty vector (●), or strain DS68625 with a vector expressing Hxt11(○), Hxt11 N366M (■) or Hxt11 N366T (□). Data calculated from the fermentation profiles presented in Figure 3

**Supplemental Table 1.**

**SNPs identified in the DS68625-evo strain by genome sequencing**

**Supplementary Table 2.**

**Fermentation profiles by the selected *S. cerevisiae* strain expressing Hxt11 and the Hxt11 N366M and N366T mutants during anaerobic batch cultivation**

|  | DS68616 | DS68625 | | |
| --- | --- | --- | --- | --- |
|  | - | Hxt11 | Hxt11-N366M | Hxt11-N366T |
| Q glucose (g glucose/g cell·h)  Q xylose (g xylose/g cell·h)  Q ethanol (g ethanol/g cell·h)  Y ethanol (g ethanol/g sugars) | 1.69 ± 0.03  0.49 ± 0.01  0.67 ± 0.02  0.43 ± 0.01 | 1.74 ± 0.06  0.54 ± 0.02  0.76 ± 0.02  0.43 ± 0.01 | 1.26 ± 0.09  0.56 ± 0.08  0.69 ± 0.01  0.40 ± 0.03 | 1.23 ± 0.01  0.63 ± 0.02  0.72 ± 0.06  0.41 ± 0.02 |

Values are the calculated average of three biological triplicates of growth of the indicated strains on 7% glucose and 4% xylose. The errors indicated are the standard error of the mean..

Q: specific productivity, Y: yield.

**Supplementary Table 3.** Oligonucleotides used in hexose transporter strain construction

| Number | Primer | Sequence (5’🡪 3’) | Gene(s) |
| --- | --- | --- | --- |
| 28 | H3f | TGTACATCCGGAATTCTAGATTGGTGAGCGCTAGGAGTCACTGCC | *HIS3* |
| 29 | H3r | CTCGAGTATTTCACACCGCATATGATCCGTCG | *HIS3* |
| 201 | Hx2uf | GACTAGTACCGGTGTTTTCAAAACCTAGCAACCCC | *HXT2* |
| 202 | Hx2ur | CGTACGCGTCTTCCGGAAGGGTACCATCAGATTTCATTTGACC | *HXT2* |
| 203 | Hx2df | GAAGACACTCGAGACGTCCTTTGTCTGTGAAACCAAGGGC | *HXT2* |
| 204 | Hx2dr | GTCGACGGGCCCTTATGTTGGTCTTGTTTAGTATGGCCG | *HXT2* |
| 205 | Hx3uf | AAGCGGCCGCACTAGTACCGGTGAAACAACTCAATAACGATGTGGGAC | *HXT3* |
| 206 | Hx3ur | ATCCGGACGTCTTCCTCAAGAAATCAGTTTGGGCGACG | *HXT3* |
| 210 | Hx4df | AGAAGACGCTCGAGACGTCCCTTATGGGAAGAAGGTGTTTTGCC | *HXT4* |
| 211 | Hx4dr | ATGGATCCTAGGGGTTCTTGCAGAGTAAACTGCG | *HXT4* |
| 212 | Hx5uf | AAGCGGCCGCACTAGTACATGTGAACTTGAAAACGCTCATCAAGGC | *HXT5* |
| 213 | Hx5ur | TTCGTACGCGTCTTCCGGAGTAACATGAAACCAGAGTACCACG | *HXT5* |
| 229 | Hx7df | AGAAGACCCTCGAGACGTCCGACGCTGAAGAAATGACTCACG | *HXT7* |
| 230 | Hx7dr | AGTCGACGGATCCGTAATTTTTCTTCTTTTAAGTGACGGGCG | *HXT7* |
| 243 | Gal2ufn | AAGCGGCCGCACTAGTACCGGTGATCTATATTCGAAAGGGGCGG | *GAL2* |
| 244 | Gal2urn | AACGTACGTCCGGATCATTAGAATACTTTTGAGATTGTGCGCT | *GAL2* |
| 233 | Ga2df | AGAAGACCCTCGAGACGTCTTACCTTGGAAATCTGAAGGCTGG | *GAL2* |
| 234 | Ga2dr | GTGGATCCTAGGTAAAACGGTACGAGAAAAGCTCCG | *GAL2* |

**Supplementary Table 4.** Oligonucleotides used for construction hexokinase deletion strain

| Number | Primer | Sequence (5’🡪 3’) | Gene(s) |
| --- | --- | --- | --- |
| 834 | Hxk2f | GCCAGAAAGGGTTCCATGGCCGATGTGCCAAAGGAATTGATGCAACAAATCCGTCGACCTCGAGTACCGTTCG | *HXK2* |
| 835 | Hxk2r | GCCAGAAAGGGTTCCATGGCCGATGTGCCAAAGGAATTGATGCAACAAATCCGTCGACCTCGAGTACCGTTCG | *HXK2* |
| 838 | Glk1f | ATGTCATTCGACGACTTACACAAAGCCACTGAGAGAGCGGTCATCCAGGCCCGTCGACCTCGAGTACCGTTCG | *GLK1* |
| 839 | Glk1r | CAATCTTCAAGTGCACCTTCCTCTCACCCTCGGCACCCAAGGGTGACAAGCCGGATCCTACCGTTCGTATAGC | *GLK1* |
| 846 | Hxk1f | ATGGTTCATTTAGGTCCAAAGAAACCACAGGCTAGAAAGGGTTCCATGGCCGGATCCACTAGCATAACTTCG | *HXK1* |
| 847 | Hxk1r | ATGGTTCATTTAGGTCCAAAGAAACCACAGGCTAGAAAGGGTTCCATGGCCGGATCCACTAGCATAACTTCG | *HXK1* |
| 848 | Gal1f | ATGACTAAATCTCATTCAGAAGAAGTGATTGTACCTGAGTTCAATTCTAGCGGATCCACTAGCATAACTTCG | *GAL1* |
| 849 | Gal1r | TTATAATTCATATAGACAGCTGCCCAATGCTGGTTTAGAGACGATGATAGTTGGGCCGCCAGTGTGATGG | *GAL1* |

**Supplementary** **Table 5.** **Oligonucleotides used in qPCR.**

| Name | Sequence (5’ 🡪 3’) |
| --- | --- |
| ActinF  ActinR  HXT8F  HXT8R  HXT9F  HXT9R  HXT10F  HXT10R  HXT11F  HXT11R  HXT12F  HXT12R  HXT13F  HXT13R  HXT14F  HXT14R  HXT15F  HXT15R  HXT16F  HXT16R  HXT17F  HXT17R | GGATTCTGAGGTTGCTGCTTTGG  GAGCTTCATCACCAACGTAGGAG  GTACTACTATCTTCAAATCTGTCGG  CTTGTGACGCCAACGGAGGCG  CCATTGAGAGGTTTGGACGCCG  ACACAATCATACAGTTACCGGCG  GGAATGCAAGACTCTTTCGAGAC  CTAGTGACGCCAACGGTGGCG  GCCACTCAATGGAGAGTCGGC  CAACTAGCAAGGCTGGATCGTC  CACCATCTTCAAATCTGTCGGTC  CAATCATACAGTTACCGGCACCC  CCCTCATGGCCAGGACGGTC  TTGCCATAACCAGTTGCATGCAG  GCCTTAGTAGTGTACTGCATCGGT  TGATACGTAGATACCATGGAGCC  GAGGCCTGTGTCTCCATCGCC  CACAAGAATACCTGTGATCAAACG  CAAGGAAGTATAGTAATACTGCGC  TTGGCGATGGAGACACAGGCC  TAACACTGCACAATGGAGAGTCC  TGAGTACCCATGGATCCTCTGG |

**Supplementary Table 6.** **Primers used for saturation mutagenesis of HXT11**

| Name | Sequence (5’ 🡪 3’) |
| --- | --- |
| F HXT11 XbaI  R HXT11 BamHI  F HXT11 366NNN  R HXT11 366NNN  F HXT11 N366F  R HXT11 N366F  F HXT11 N366E  R HXT11 N366E  F HXT11 N366K  R HXT11 N366K  F HXT11 N366M  R HXT11 N366M  F HXT11 N366W  R HXT11 N366W  F HXT11 N366Y  R HXT11 N366Y | GGCCTCTAGAATGTCAGGTGTTAATAATACATCCGC CGATGGATCCTCAGCTGGAAAAGAACCTCTTGTAAATTG CGGTGTGGTTnnnTTTTTCTCTTCATTC  GAATGAAGAGAAAAA nnnAACCACACCG  CGGTGTGGTTtttTTTTTCTCTTCATTC  GAATGAAGAGAAAAAaaaAACCACACCG  CGGTGTGGTTgagTTTTTCTCTTCATTC  GAATGAAGAGAAAAActcAACCACACCG  CGGTGTGGTTaaaTTTTTCTCTTCATTC  GAATGAAGAGAAAAAtttAACCACACCG  CGGTGTGGTTatgTTTTTCTCTTCATTC  GAATGAAGAGAAAAAcatAACCACACCG  CGGTGTGGTTtggTTTTTCTCTTCATTC  GAATGAAGAGAAAAAccaAACCACACCG  CGGTGTGGTTtatTTTTTCTCTTCATTC  GAATGAAGAGAAAAAataAACCACACCG |

n is any nucleotide
